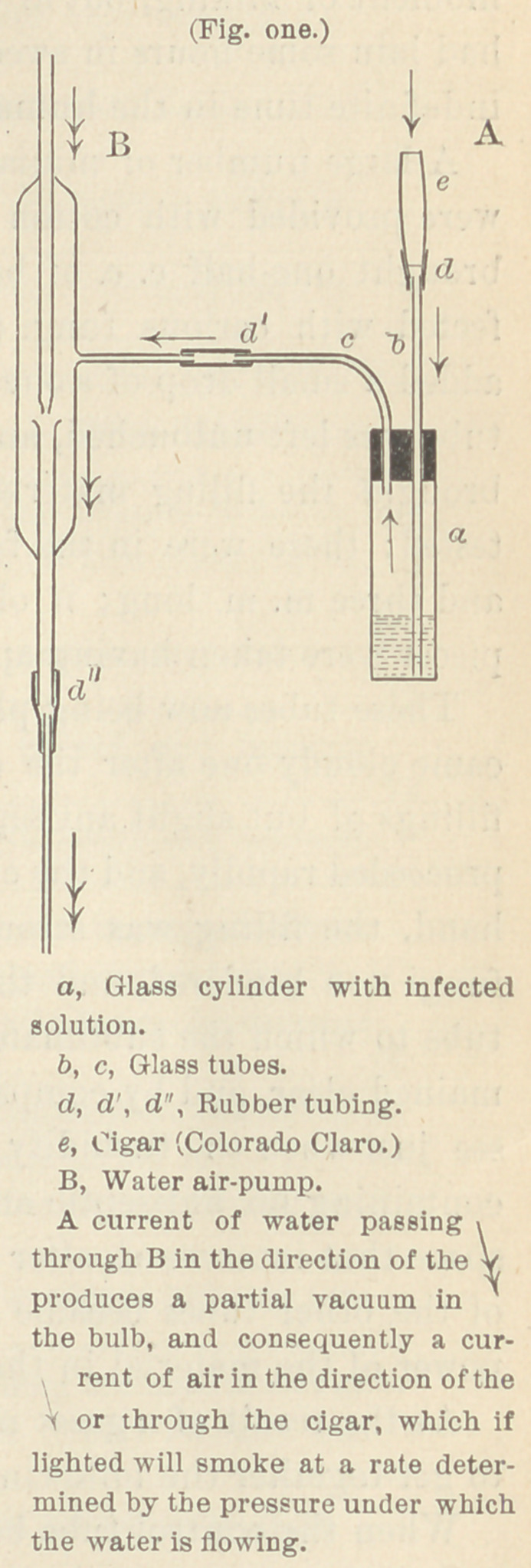# Fermentation in the Human Mouth

**Published:** 1884-06

**Authors:** W. D. Miller

**Affiliations:** Berlin, Germany


### Independent Practitioner.

VOL. V. JUNE, 1884. No. 6.

### Original Communications.

### FERMENTATION IN THE HUMAN MOUTH.

### THE INFLUENCE OF ANTISEPTICS, FILLING MATERIALS, ETC., UPON THE FUNGI OF DENTAL CARIES.

BY DR. W. D. MILLER, BERLIN, GERMANY.

(Continued from page 233.)

Having established upon an experimental and scientific basis the fact that caries of the teeth is, to a certain extent, the direct result of the action of ferment acid or acids* upon the tissue of the tooth, followed, particularly in the case of the dentine, by the action of the ferment organisms themselves upon the decalcified tissue, it becomes a matter of the first importance to determine, first, by what means we may counteract the action of the acids or prevent their production; second, by what means we may save the already decalcified dentine from complete destruction.

Evidently there are three methods by which the desired end may be partially obtained:

1. By repeated, thorough, systematic cleansing of the oral cavity and the teeth, we may so far reduce the amount of fermentable substances in the mouth and the number of ferment organisms, as to materially diminish the production of acid. This is so self-evident that it needs no further comment.

* The chief work in the production of caries is performed by lactic acid; other acids are only auxiliary factors.

2. By the repeated application of alkaline substances we may, to a certain extent, neutralize the acids before they have acted upon the teeth to any considerable degree.

3. By a proper and intelligent use of antiseptics we may destroy the organisms themselves, or at least render them inactive. It is this method which is especially applicable in the second stage of dental caries (*i. e.*, the stage which follows the decalcification), and to which we will here give exclusive attention. We must, however, constantly bear in mind that by whatever method we proceed, a previous thorough cleansing of the teeth is absolutely indispensable. There is no known solution, alkaline or antiseptic, applicable in the human mouth, which will penetrate between the teeth or to the bottom of fissures and cavities, when these are filled with food, in sufficient quantity to have any appreciable effect. Therefore, before all antiseptics or alkaline washes come the toothbrush, toothpick, and floss silk.

In my experiments for determining the action of various antiseptics upon the fungi of tooth caries, it appeared to me that by allowing the antiseptic to act upon the fungi in their natural medium, saliva, I could obtain results of more practical value than by experimenting upon them in artificial solutions, and in pure cultures, neither of which ever occurs in the human mouth. Furthermore, since the fungi can attack the teeth only after a partial decalcification, we have in the first place to demand of an antiseptic, not so much that it destroys the fungi, as that it prevents the production of acid by them.* Consequently, if an acid reaction failed to appear in a solution of saliva and sugar to which a certain antiseptic had been added, as soon as in a like solution to which no antiseptic had been added (control), it was taken as evidence of the activity and value of the antiseptic used. This method could of course be used only with substances having a neutral reaction. The solutions were also subjected to a microscopic examination, to render the evidence doubly sure.

* The production of acid may be taken as synonymous with the development of the fungi, though the failure of the acid reaction to appear after a certain length of time does not necessarily indicate that the fungi have been *devitalized.*

In the following table I have indicated the percentage of each antiseptic experimented upon which must be present in a sweetened-saliva solution, to prevent the appearance of an acid reaction in twenty-four hours, or in case of alkaline or acid antiseptics, to prevent the development of the characteristic fungi in the same time.

For example, if to 100,000 parts of sweetened saliva we add one part of bichloride of mercury, the solution will not be found acid after the lapse of twenty-four hours, even though the control became sour in four or five hours. If we add only one part to 500,000, the acid reaction will appear somewhat later than in the control.

This table is designed to show the comparative strength of the antiseptics most commonly used. The action of the antiseptics having an acid or alkaline reaction upon the fungi, was determined by the use of the microscope alone.

| | Production of Acid (Development of Fungi) | |
|---|---|---|
| | Prevented. | Retarded. |
| Bichloride of mercury | 1–100,000 | 1–500,000 |
| Nitrate of silver | 1–50,000 | 1–100,000 |
| Iodoform | 1–5,000 | 1–10,000 |
| Naphthaline | 1–4,000 (?) | 1–9,000 |
| Iodine | 1–6,000 | 1–15,000 |
| Oil of mustard | 1–2,000 | 1–5,000 |
| Permanganate of potas | 1–1,000 | 1–2,000 |
| Eucalyptus oil | 1–600 | |
| Carbolic acid | 1–500 | 1–1,000 |
| Hydrochloric acid | 1–500 | 1–1,000 |
| Phenylic acid | 1–200 | 1–500 |
| Liquid of Agate Cement | 1–250 | |
| Liquid of Excelsior Cement | 1–225 | |
| Lactic acid | 1–125 | 1–250 |
| Carbonate of sodium | 1–100 | 1–200 |
| Salicylic acid (Conc. alcohol sol.) | 1–75 | 1–125 |
| Alcohol | 1–10 | 1–20 |

The experiments show that bichloride of mercury is about two hundred times as powerful as carbolic acid, and demonstrate very clearly the mistake of substituting weak solutions of this antiseptic

(1–1,000, as I have seen recommended) for concentrated carbolic acid. One one-thousandth is only one-fifth as powerful as pure carbolic acid, which in many cases may be used with impunity. It is consequently useless to attempt to introduce the sublimate solution for the purpose of sterilizing root canals, cavities before filling, etc., unless we may use at least a one-half per cent., if not a one per cent. solution. I see no reason, however, why this may not be done. In a few cases I have used a one per cent. solution for treating root canals, and do not hesitate, particularly with the rubber dam adjusted, to wipe out cavities before filling with a two per cent. solution, and see no possible evil which could result from it. A well-known physiologist in Berlin has told me that he uses a one per cent. solution in his own mouth for aphthæ, and with excellent results. We should not, however, overlook the fact that a one per cent. sublimate solution is only one-fifth as powerful as pure iodoform.

As a mouth wash I have frequently used a one-tenth per cent. (1–1,000) solution myself, and have seen no bad results from it; I would not, however, recommend it to my patients in this strength. It has, besides, for me, an exceedingly disagreeable and lasting taste, which it is difficult to disguise, and produces an immediate increased secretion of saliva and mucus, which is very annoying. A one-fiftieth per cent. solution (1–5,000) may eventually be brought into use; in this concentration it is four times as powerful as a one per cent. solution of carbolic acid. The very high antiseptic power of nitrate of silver is particularly noteworthy. Why may it not be employed in place of the much more dangerous mercuric chloride?

The action of tobacco upon the fungi is worthy of notice. Five grammes of old Virginia plug were boiled fifteen minutes in fifty c. c. of water, the loss by evaporation being constantly replaced; the decoction was then filtered, and a portion added to an equal volume of saliva with sugar. This produced a mixture scarcely stronger than that which many veteran chewers carry around in their mouths all day, and in it the fungi led only a miserable existence.

Much more remarkable, however, was the action of tobacco smoke upon the fungi; the smoke from the first third or last quarter of a

Colorado Claro cigar being found amply sufficient to sterilize ten c. c. of a beef-extract-sugar solution, previously richly infected with caries fungi.

The apparatus used for this experiment (see figure one) explains itself. A current of water passing through the part B in the direction of the ↓ produces a current of air through the part A, in the direction of the ↓ which draws the smoke from a lighted cigar through the solution. The rate at which the cigar smokes may be regulated at will by the cock of the hydrant.

(Fig. one.)

*a*, Glass cylinder with infected solution.

*b*, *c*, Glass tubes.

*d*, *d'*, *d''*, Rubber tubing.

*e*, Cigar (Colorado Claro.)

B, Water air-pump.

A current of water passing through B in the direction of the ↓ produces a partial vacuum in the bulb, and consequently a current of air in the direction of the ↓ or through the cigar, which if lighted will smoke at a rate determined by the pressure under which the water is flowing.

In consideration of the strong antiseptic power of tobacco smoke, we might be inclined to infer that tobacco smokers should never suffer from caries of the teeth; it is evident, however, that there are very many points in the dental arch to which the smoke never penetrates.

In the preparation of cavities for inserting fillings, it is naturally often next to impossible to remove all the carious dentine, and in all such cases it is especially desirable that the filling material itself should possess antiseptic properties, since we, in using such a material, not only destroy those organisms existing in the carious tissue, but the material, if it remains permanently antiseptic, retards the working of the ferment organisms from without, and the appearance of secondary decay. We need, therefore, a material for filling which is not only antiseptic at the time of insertion, but which remains permanently so after being inserted.

I have endeavored to determine the relative antiseptic power of different filling materials (cements, amalgams, etc.), not only at the moment of mixing, but after they were thoroughly dry, after they had lain some hours in sweetened saliva, and after they had been an indefinite time in the human mouth.

A large number of miniature test-tubes (homeopathic pill-tubes) were provided with cotton stoppers, and sterilized. Into each was brought one-half c. c. of beef-extract-sugar solution, previously infected with carious fungi (pure culture). To the first tube was added a small drop of a one per cent. sublimate solution; the second tube was left untouched, and into the third, fourth, fifth, etc., were brought the filling materials whose antiseptic virtues were to be tested; these were in the form of cylinders two m. m. in diameter, and three m. m. long; if old fillings from the mouth were used, pieces were taken having approximately the same size.

These tubes now being placed in the incubator, their contents became cloudy one after the other. In those tubes which contained fillings of but slight antiseptic power, the development of the fungi proceeded rapidly, and the cloudiness soon appeared. If, on the other hand, the filling was strongly antiseptic, the development of the fungi was hindered, and the cloudiness appeared later. The first tube to which the sublimate solution had been added of course remained clear, and by comparing the others with this it was easy to see just when the turbidity began to show itself; the second tube, containing no antiseptic and no filling, served as control, and the space that intervened after the control became turbid till any one of the other tubes became turbid, was a measure of the antiseptic power of the material in that tube.

As the result of a great number of experiments, I have been able to get together the following table:

When the control tube becomes turbid in 5 hours, then:

| | | |
|---|---|---|
| A tube containing an old oxy-phosphate filling becomes turbid in... | 5 | hours. |
| " " " oxy-chloride " " " "... | 5 | " |
| " " a gold cylinder becomes turbid in............... | 5 | " |
| " " a Hill's stopping cylinder becomes turbid in..... | 5 | " |
| " " an amalgam cylinder (kept 12 hours in saliva) becomes turbid in.......................... | 5½ | " |

A tube containing an agate cylinder (kept 12 hours in saliva) becomes turbid in.......... $5\frac{1}{4}$ hours.

" " an old amalgam filling becomes turbid in ...... $5\frac{3}{10}$ "

" " an amalgan cylinder (mixed dry) becomes turbid in.......... $5\frac{3}{8}$ "

" " an amalgam cylinder (mixed wet) becomes turbid in .......... $5\frac{1}{2}$ "

" " an oxy-phosphate cylinder (12 hours in saliva) becomes turbid in.......... $5\frac{1}{2}$ "

" " an amalgam cylinder (12 hours old) becomes turbid in.......... $5\frac{2}{3}$ "

" " an old filling of tin and gold becomes turbid in.. $5\frac{4}{5}$ "

" " an oxy-phosphate cylinder (12 hours old) becomes turbid in.......... 6 "

" " an agate cylinder (12 hours old) becomes turbid in. $6\frac{1}{4}$ "

" " an iodoform cement cylinder (12 hours in saliva) becomes turbid in.......... $6\frac{2}{5}$ "

" " a pyrophosphate cylinder (mixed dry) becomes turbid in.......... $7\frac{1}{8}$ "

" " a pyrophosphate cylinder (mixed wet) becomes turbid in.......... $7\frac{3}{8}$ "

" " an oxy-chloride cylinder (12 hours old) becomes turbid in.......... 9 "

" " a piece of dentine from a tooth impregnated by a copper amalgam filling becomes turbid in. 11 "

" " an iodoform cement cylinder (12 hours old) becomes turbid in.......... 12 "

" " an iodoform cement cylinder (fresh) becomes turbid in ? "

" " a globule of mercury becomes turbid in.......... — "

" " a cylinder of black oxide of mercury becomes turbid in.......... — "

" " a cylinder of any copper amalgam becomes turbid in.......... — "

" " any old copper amalgam filling becomes turbid in — "

" " a cylinder of oxy-chloride (fresh) becomes turbid in.......... — "

The (—) signifies that the solution remained permanently clear.

We see from these results that the only filling at present in use which exerts a continual anti-ferment* action upon the walls of the

* I use the terms anti-ferment and anti-septic interchangeably, though the former is, perhaps, preferable, since we are treating of ferment, and not septic organisms.

tooth and its immediate surroundings, is the old copper amalgam; not only that, but the very substance of the tooth containing such a filling itself becomes antiseptic, a piece of bluish or bluish-green dentine from such a tooth very powerfully retarding the development of the fungi, and, indeed, in two cases completely destroying them. Secondary decay in such a case would be next to impossible, where anything like cleanliness was observed.

This result is well supported by observations which I have had abundant opportunity to make for the last five years, here where this material is so extensively used, and I do not hesitate to say that if our only object is to check the destruction of tissue by caries, there is no material at present in use with which this object may be so surely accomplished as with a good copper amalgam. It is a material, however, which I have never used, though I am not aware of any bad effect produced by it beyond the discoloration of the tooth. Skogsberg's iodoform cement came into my hands too late to complete the experiments with it. It has undoubtedly strong antiseptic properties, which it does not completely lose even when exposed to the saliva, and might no doubt be used to great advantage as a foundation for permanent fillings. Old fillings of tin and gold possess slight antiseptic power, still less (almost zero) old amalgam fillings (not copper). The very inconsiderable power of amalgams to prevent the development of ferment fungi is a source of some surprise, since we have been accustomed to look upon them as very active in this respect. It is probably a mistake to attribute the hardening of dentine under amalgam fillings to the antiseptic action of the amalgam, since, in the first place, it possesses this power to but a slight degree, and in the second place the hardening may take place under fillings of gutta percha equally well. If we dry the cavity but indifferently well, and then choose a piece of gutta percha which we think will about fit the cavity, warm it and stuff it into the cavity, we of course can expect only bad results. If we proceed as follows we will obtain excellent results, as I have seen time and again: Adjust the dam, excavate carefully, especially the margins, wash with a strong antiseptic, dry thoroughly with bibulous paper and then with the hot-air syringe, till the surface of the dentine becomes whitish, paint with a thin solution of copal varnish, dry again with warm air, then put in the gutta percha in small pieces, one after the other, being *sure* that each piece sticks to its place, especially along the margin, just as if you were making a filling of gold. A piece which has once moved in its place must not be allowed to remain, as a leak will be the result. Remove such a filling after two years, and the cavity will often be found in an excellent condition for a gold filling.

The oxy-chlorides when first mixed, are powerfully antiseptic, but soon lose their energy when exposed to the action of saliva.

The oxy-phosphates are very much inferior to the oxy-chlorides in antiseptic power, and should never be used in cavities where there is much soft dentine. This conclusion is borne out by my own experience in practice, and by that of others with whom I have conversed on the subject. Dr. Paetsch first called my attention to the disastrous results of such a practice, and his testimony was confirmed by that of Dr. F. P. Abbott, and others.

It must not be expected that the results given in the above table are absolutely free from error. The experiment is attended with more difficulties than are at first sight apparent; especially does the sterilization of the filling materials themselves involve much time and labor, and the results are not always constant; this was especially the case with iodoform cement. Amalgams and phosphates gave quite constant results. The tests with some of the materials were made over twenty-five times; with others, such as copper amalgams, where there was no doubt as to the result, only a few experiments were made.

Caries of the teeth, except in the later or last stage, is the result of a ferment process, and the organisms found in the deeper parts of decaying dentine, which I have isolated and obtained in pure culture, are ferment organisms. The decomposition of the pulp and contents of the root canal, attended by bad-smelling products, is, on the other hand, a putrefactive process, in which entirely different species of fungi are concerned. Whether or not the results which I have obtained for the fungi of caries would apply equally well to those putrefactive fungi, is a question which can be settled only by experiment upon pure cultures of the same.

Although I have now, as I think will be granted, established upon a sure basis a fact that caries of the teeth may result directly from the action of acid-producing fungi in the presence of fermentable carbo-hydrates, the conclusion would hardly be justifiable that, by keeping the mouth constantly and perfectly free from all fermentable substances, or by repeated application of antacids or antiseptics to all parts of the teeth, or by all these means together, we could ever banish dental caries from the oral cavity. A most powerful influence, which we do not well understand, is exerted by the nutritive processes in the teeth themselves.

I am assured by men who have grown old in the practice of dentistry, that mouths which have long been under their observation, and which practically have been completely free from caries for years, at once, on account of some sudden change of health, show a general breaking down or crumbling of the teeth, *en masse*, in the space of a few weeks. It has also been my experience that patients who have been dismissed by their dentists in America, with the assurance that according to previous experience their dentures would require no treatment for one or two years, have come to me a few weeks later with teeth looking as though they had not been under the hands of a dentist for years. Some say the ocean voyage spoiled their teeth; others attribute it to a change in the climate, food, health, etc.

At any rate, we have here a cause which lies without the domain of both bacteria and acids (either ferment or otherwise). The lime-salts of the teeth are supposed to form, with the organic matter of the tooth, a definite chemical compound, and it is probably due to this fact that simple salts of lime are so much more readily soluble in weak acids than pulverized tooth-bone, or that the tartar upon the teeth is so much more easily soluble than the teeth themselves; so that when any one rinses his mouth with vinegar, and afterwards finds lime in the vinegar, we know that the lime, in by far the greater part, if, indeed, we may not say altogether, came from the tartar. Now, though there is no positive evidence for the supposition, it is certainly not altogether improbable that, as a consequence of certain derangements in the nutritive functions of the teeth resulting from a change of health, etc., etc., a dissolution of the affinity between the lime-salts and the organic matter may take place, thus setting free the easily soluble lime-salts, which are then carried away in solution or washed out mechanically.

This is a supposition only, which I bring forward because facts in this case are absolutely wanting. If it should, perchance, contain a trace of truth, then adult and pulpless teeth should be less subject to these *sudden* attacks of caries than young teeth with living pulps.

There still remains much hard work to be done, before the subject of dental caries may be dismissed as having received a final solution *in all its different phases.* There are men enough in the profession, however, who are willing to work, and who do not shrink from the tasks yet to be performed.

## Figures and Tables

**Fig. one. f1:**